# Post-anesthesia care unit hypotension in low-risk patients recovering from non-cardiac surgery: a prospective observational study

**DOI:** 10.1007/s10877-024-01176-9

**Published:** 2024-05-17

**Authors:** Moritz Flick, Anneke Lohr, Friederike Weidemann, Ashkan Naebian, Phillip Hoppe, Kristen K. Thomsen, Linda Krause, Karim Kouz, Bernd Saugel

**Affiliations:** 1 Department of Anesthesiology, Center of Anesthesiology and Intensive Care Medicine, University Medical Center Hamburg-Eppendorf, Hamburg, Germany; 2 Institute of Medical Biometry and Epidemiology, University Medical Center Hamburg-Eppendorf, Hamburg, Germany; 3 Outcomes Research Consortium, Cleveland, OH USA

**Keywords:** Blood pressure, Finger-cuff, Hemodynamic monitoring, Non-invasive, Postoperative hypotension

## Abstract

Intraoperative hypotension is common and associated with organ injury. Hypotension can not only occur during surgery, but also thereafter. After surgery, most patients are treated in post-anesthesia care units (PACU). The incidence of PACU hypotension is largely unknown – presumably in part because arterial pressure is usually monitored intermittently in PACU patients. We therefore aimed to evaluate the incidence, duration, and severity of PACU hypotension in low-risk patients recovering from non-cardiac surgery. In this observational study, we performed blinded continuous non-invasive arterial pressure monitoring with finger-cuffs (ClearSight system; Edwards Lifesciences, Irvine, CA, USA) in 100 patients recovering from non-cardiac surgery in the PACU. We defined PACU hypotension as a mean arterial pressure (MAP) < 65 mmHg. Patients had continuous finger-cuff monitoring for a median (25th percentile, 75th percentile) of 64 (44 to 91) minutes. Only three patients (3%) had PACU hypotension for at least one consecutive minute. These three patients had 4, 4, and 2 cumulative minutes of PACU hypotension; areas under a MAP of 65 mmHg of 17, 9, and 9 mmHg x minute; and time-weighted averages MAP less than 65 mmHg of 0.5, 0.3, and 0.2 mmHg. The median volume of crystalloid fluid patients were given during PACU treatment was 200 (100 to 400) ml. None was given colloids or a vasopressor during PACU treatment. In low-risk patients recovering from non-cardiac surgery, the incidence of PACU hypotension was very low and the few episodes of PACU hypotension were short and of modest severity.

## Introduction

In patients having surgery with general anesthesia, hypotension is common and associated with organ injury [1–6]. Hypotension can not only occur during surgery [2, 3, 7], but also thereafter [8, 9].

Patients who are not admitted to high-dependency or intensive care units after surgery are monitored and treated in post-anesthesia care units (PACU) before they are transferred to general wards. During this early postoperative recovery in the PACU, patients may be at particular risk of developing hypotension, e.g. due to prolonged effects of anesthetic drugs or postoperative bleeding [10]. However, the incidence, duration, and severity of PACU hypotension is largely unknown – presumably in part because arterial pressure is usually monitored intermittently, often only at 15-minute intervals, in PACU patients [11].

We therefore aimed to evaluate the incidence, duration, and severity of PACU hypotension in low-risk patients recovering from non-cardiac surgery using blinded continuous arterial pressure monitoring with a finger-cuff.

## Materials and methods

Our study was a pre-specified observational add-on study of the single-center randomized DETECT trial [12]. DETECT showed that continuous finger-cuff – compared to intermittent oscillometric – arterial pressure monitoring helps clinicians reduce hypotension during induction of anesthesia and during non-cardiac surgery [12]. This add-on study was approved by the ethics committee (Ethikkommission der Ärztekammer Hamburg, Hamburg, Germany, registration number PV7361) a month after DETECT had started and was performed between April and October 2021 at the University Medical Center Hamburg-Eppendorf, Hamburg, Germany. All patients provided written informed consent. The study was registered in the German Register for Clinical Studies (Registration: DRKS00024881). Our report follows the Strengthening the Reporting of Observational Studies in Epidemiology (STROBE) guidelines [13].

### Patients

DETECT included 242 patients ≥ 45 years old who had elective non-cardiac surgery with general anesthesia and planned intermittent oscillometric arterial pressure monitoring with an upper-arm cuff. Patients who needed intraarterial arterial pressure monitoring or who had a systolic arterial pressure difference of more than 20 mmHg between the right and left arm were excluded. Other exclusion criteria were emergency surgery, pregnancy, and heart rhythms other than sinus rhythm.

Patients in DETECT were randomized to unblinded continuous finger-cuff arterial pressure monitoring or to intermittent oscillometric arterial pressure monitoring with blinded continuous finger-cuff arterial pressure monitoring during induction of anesthesia and during surgery. Patients randomized to intermittent oscillometric arterial pressure monitoring with blinded continuous finger-cuff arterial pressure monitoring were eligible for participation in this add-on study.

### Postoperative management

After emergence from general anesthesia, patients were transferred to the PACU. Patients were monitored and treated in the PACU until clinicians considered them fit enough for transfer to the general ward. Per clinical routine, arterial pressure was monitored at 5-minute intervals using oscillometry. Postoperative hemodynamic management aimed at keeping mean arterial pressure (MAP) above 65 mmHg using fluids and norepinephrine.

### Study measurements

Study measurements for this add-on study started when patients had arrived in the PACU. During the PACU stay, we performed blinded continuous arterial pressure monitoring with finger-cuffs (ClearSight system; Edwards Lifesciences, Irvine, CA, USA) positioned on the third or fourth finger of the arm contralateral to the oscillometric cuff [12]. The ClearSight system was zeroed using a heart reference sensor attached to the patient’s chest at the level of the right atrium. We extracted arterial pressure measurements from the ClearSight system as 20-second averages.

Before analysis, we excluded artifactual arterial pressure measurements as follows: (1) arterial pressure values documented as artifacts by study personnel; (2) systolic arterial pressures > 280 mmHg or < 30 mmHg; (3) systolic arterial pressures below the diastolic arterial pressure plus 5 mmHg; or (4) diastolic arterial pressures > 150 mmHg or < 10 mmHg. We replaced excluded and missing 20-second averaged arterial pressure values by the mean of the closest 20-second averaged arterial pressure values.

### Hypotension exposure

We quantified PACU hypotension using continuous finger-cuff arterial pressure monitoring. We defined PACU hypotension as a MAP < 65 mmHg. We assessed the proportion of patients who had a MAP < 65 mmHg for at least one consecutive minute. We further quantified the duration and severity of PACU hypotension by calculating the cumulative duration patients had a MAP < 65 mmHg (mins), the area under a MAP of 65 mmHg (mmHg x min), and the time-weighted average MAP less than 65 mmHg (mmHg). We calculated the same measures for MAP thresholds of 70 mmHg and 80 mmHg.

### Statistical analysis

Categorical data are presented as absolute number (percentage), continuous data as mean (standard deviation) or median (25th percentile, 75th percentile). Statistical analyses were performed with R version 4.1.2. (R Core Team (2021). R: A language and environment for statistical computing. R Foundation for Statistical Computing, Vienna, Austria. URL https://www.R-project.org/).

Based on previous studies investigating postoperative hypotension on general wards, we estimated that the incidence of PACU hypotension, defined as a MAP < 65 mmHg for at least one minute, would be about 15% [14, 15]. Based on this assumption, a sample size of 100 patients would produce a two-sided 95% confidence interval with a width equal to 14.9% [16].

## Results

We included 100 patients in this study, and all were included in the analysis (Table 1). Patients had continuous finger-cuff monitoring for a median (25th percentile, 75th percentile) monitoring time per patient of 64 (44 to 91) minutes. Of 20,737 finger-cuff arterial pressure measurements, we replaced 117 missing (0.6%) and 5 artefactual (0.002%) measurements.

**Table 1:** Demographics and medical history (*n* = 100 patients)

Age, years		62 (11)
Female, n		36 (36%)
Weight, kg		85 (17)
Height, cm		175 (9)
Body mass index, kg/m^2^		26 (5)
American Society of Anesthesiologists physical status (I; II; III), n		13 (13%); 73 (73%); 14 (14%)
**Medical History**		
	Chronic arterial hypertension, n	44 (44%)
	Chronic heart failure	0 (0%)
	Coronary artery disease, n	6 (6%)
	Chronic kidney disease, n	1 (1%)
	Chronic obstructive pulmonary disease, n	4 (4%)
	Diabetes mellitus, n	6 (6%)
	Cerebrovascular disease, n	7 (7%)
	Revised cardiac risk index, (score 0; 1; 2), n	76 (76%); 22 (22%); 2 (2%)
**Type of surgery**		
	Ear, nose, and throat	16 (16%)
	General	9 (9%)
	Gynecology	11 (11%)
	Neurology	6 (6%)
	Oral and maxillofacial	13 (13%)
	Trauma	5 (5%)
	Urology	40 (40%)

Data are presented as mean (standard deviation) or absolute number (percentage)

**Fig. 1:**
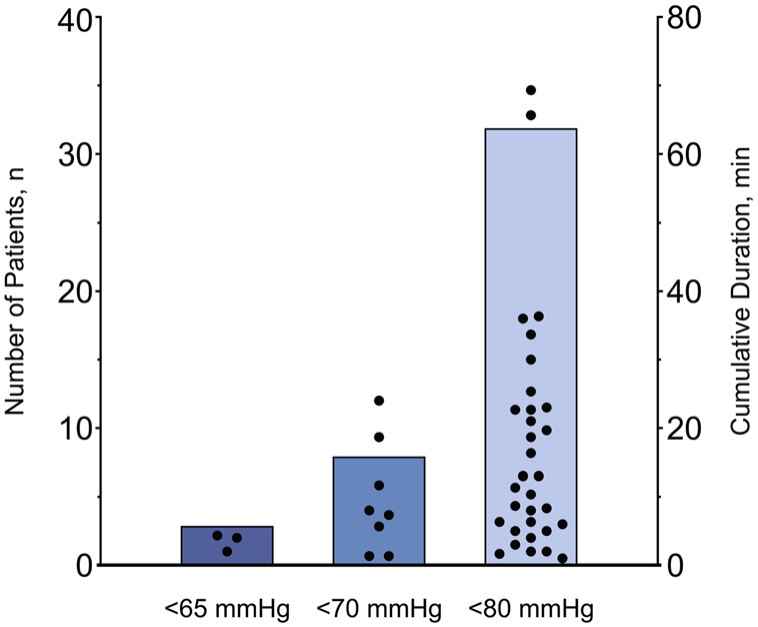
Number of patients below mean arterial pressure thresholds for at least one minute (vertical bars) and cumulative duration below thresholds of individual patients (black dots)

Only three patients (3%) had PACU hypotension (MAP < 65 mmHg) for at least one consecutive minute (Fig. 1). These three patients had 4, 4, and 2 cumulative minutes of PACU hypotension; areas under a MAP of 65 mmHg of 17, 9, and 9 mmHg x minute; and time-weighted averages MAP less than 65 mmHg of 0.5, 0.3, and 0.2 mmHg.

Eight patients (8%) had a MAP < 70 mmHg for at least one minute. In these eight patients, the median duration patients had a MAP < 70 mmHg was 8 (2 to 17) cumulative minutes; the median area under a MAP of 70 mmHg was 42 (32 to 59) mmHg x minute; and the median time-weighted average MAP less than 70 mmHg was 0.9 (0.3 to 1.5) mmHg.

Thirty-two patients (32%) had a MAP < 80 mmHg for at least one minute. In these 32 patients, the median duration patients had a MAP < 80 mmHg was 12 (5 to 23) cumulative minutes; the median area under a MAP of 80 mmHg was 43 (23 to 130) mmHg x minute; and the median time-weighted average MAP less than 80 mmHg was 0.7 (0.4 to 1.6) mmHg.

The median volume of crystalloid fluid that patients were given during PACU treatment was 200 (100 to 400) ml. None was given colloids or a vasopressor during PACU treatment. Three patients were given nifedipine for hypertension, and 5 were given clonidine (none of those patients developed PACU hypotension).

## Discussion

We used blinded continuous non-invasive arterial pressure monitoring to investigate the incidence, duration, and severity of PACU hypotension in low-risk patients recovering from non-cardiac surgery. There was almost no PACU hypotension, and what hypotension we observed was short and of modest severity.

A recent analysis of 104,875 patients recovering from low- to high-risk non-cardiac surgery reported a 12% incidence of PACU hypotension [11]. This previous analysis also defined PACU hypotension as a MAP < 65 mmHg but reports the incidence of any MAP < 65 mmHg – while the 3% incidence of PACU hypotension we observed refers to hypotensive events lasting at least one consecutive minute. Furthermore, the previous analysis – compared to our study – included sicker patients (40% were classified as American Society of Anesthesiologists physical status (ASA) class III, and 4% ASA class IV) who may have a higher risk for postoperative hypotension. Additionally, patients stayed in the PACU for several hours, thus increasing exposure time, whereas our patients usually were transferred to a general ward after about an hour.

In our PACU, arterial pressure is routinely measured every 5 minutes, which may be more frequent than in other hospitals where arterial pressure often is only measured every 15 mins [11]. One thus may speculate that PACU hypotension was sparse because it was quickly identified and treated. However, patients were given no vasopressors and only small amounts of crystalloids while in the PACU. Close monitoring and early intervention were therefore not the reason that hardly any PACU hypotension occurred. Patients in our study simply did not develop PACU hypotension – presumably because we included relatively healthy patients who had low- to moderate-risk surgery.

It remains unknown whether hypotension harm thresholds differ between awake patients and patients having surgery with general anesthesia in whom energy expenditure is about a quarter lower than in awake patients [17, 18]. Large registry studies report that the intraoperative population harm threshold for organ injury is a MAP near 65 mmHg [2, 4, 6, 19, 20]. We thus primarily used a MAP threshold of 65 mmHg to define PACU hypotension. However, some studies suggest that postoperative hypotension harm thresholds – in awake patients – may be higher, perhaps at a MAP near 80 mmHg [21, 22]. Naturally, more patients had MAP values below 70 mmHg and 80 mmHg than below 65 mmHg. Whether MAP values around 70–80 mmHg constitute clinically important hypotension in awake patients requires further investigation. As normal arterial pressures substantially vary among individual patients presenting for surgery [23], it seems reasonable to assume that postoperative hypotension harm thresholds also differ among individuals.

We monitored arterial pressure continuously using the finger-cuff method that is well-validated [24, 25]. We blinded clinicians to continuous arterial pressure monitoring so clinical management was exclusively guided by oscillometric measurements at 5-minute intervals. Therefore, a strength of this study is that we continuously monitored arterial pressure – and thus unlikely missed hypotension [12, 26–28] – while patients had routine care.

We deliberately included relatively healthy patients who had no indication for invasive arterial pressure monitoring with an arterial catheter and who were treated in our normal PACU after low- to-moderate-risk surgery. Naturally, our results cannot be generalized to patients with higher baseline risk or patients having major surgery, but presumably reflect the incidence, duration, and severity of hypotension in typical surgical patients. The observed incidence as well as duration and severity of PACU hypotension was lower than expected. Not all patients from the DETECT trial were recruited for this add-on study due to delays in ethics approval of this add-on study. However, it seems unlikely though that a larger sample size would have yielded in substantially different results.

## Conclusion

In low-risk patients recovering from non-cardiac surgery, the incidence of PACU hypotension was very low and the few episodes of PACU hypotension were short and of modest severity. Our results cannot be generalized to patients with higher baseline risk or patients having major surgery. The incidence and clinical importance of PACU hypotension in high-risk patients recovering from major surgery warrants further investigation.

## Data Availability

No datasets were generated or analysed during the current study.
